# Investigating the Role of HOTAIR and MALAT1 Long Noncoding RNAs and Their Relations With Bone Marrow Environment in Acute Myeloid Leukemia Subtypes: Biomarkers and Treatment Response

**DOI:** 10.1155/ah/3459924

**Published:** 2025-10-08

**Authors:** Hero Hamad, Goran Othman, Ranan Kardagh

**Affiliations:** ^1^Department of Medical Laboratory Technology, Erbil Health and Medical Technical College, Erbil Polytechnic University, Erbil, Iraq; ^2^Department of Pathology and Hematology, College of Medicine, Hawler Medical University, Erbil, Iraq; ^3^Department of Medical Laboratory Technology, Al-Qalam University College, Kirkuk, Iraq

**Keywords:** AML, bone marrow environment, HOTAIR, LncRNA, MALAT1

## Abstract

Long noncoding RNAs have recently emerged as major players in cancer by operating through complex structural and functional diversity in a wide range of cellular processes. Among these, certain lncRNAs, including MALAT1 and HOTAIR, have been in the limelight concerning AML for their important roles played in regulating gene expression that in turn influence the disease course of AML. This review summarizes the structure and classification of lncRNAs, mechanisms of action regarding cancer biology, and how lncRNAs such as MALAT1 and HOTAIR act as oncogenes or tumor suppressors. It also examines intricate correlations among these lncRNAs and the bone marrow microenvironment with regard to effects on AML cell proliferation, migration, and survival. In the current review, the key pathways in AML, through which MALAT1 and HOTAIR drive cellular proliferation and epigenetic processes, are discussed in detail to point out possible therapeutic targets. The interactions between MALAT1 and HOTAIR within the bone marrow microenvironment suggest the diverse involvement of lncRNAs in AML and support their applications in biomarker development and as novel avenues for targeted therapies. This review thus represents a broad overview with the intention of furthering our understanding of the lncRNA-mediated pathways in AML and their use as diagnostic and therapeutic tools.

## 1. Introduction

LncRNAs are a family of RNA molecules longer than 200 nucleotides. Although they do not code for proteins, they play important roles in transcription, post-transcriptional processing, and chromatin remodeling, among other aspects of the control of gene expression [1, 2]. In the past 10 years, lncRNAs have come to be understood as crucial regulators of a number of cellular processes, especially those related to cancer, impacting the formation, differentiation, and disease progression [3]. LncRNAs are commonly categorized as sense, antisense, bidirectional, intronic, and intergenic based on their genomic locations in relation to protein-coding genes [4]. Lacking the same selective pressure for primary sequence conservation as seen in protein-coding genes, many lncRNAs possess conserved secondary structures that offer the framework for a spectrum of function [5].

LncRNA dysregulation contributes to the different hallmarks of cancer, including metastasis, resistance to cell death, and sustained proliferative signaling [6, 7]. Considering the biological function of lncRNAs, some of them act through an oncogenic role, while others can express tumor suppressor activity. A paradigm example of an oncogenic lncRNA in multiple cancers, HOTAIR exerts its function through the reprogramming of chromatin states toward tumor suppressor gene silencing [8, 9]. LncRNAs are also implicated in the processes of metastasis and the tumor microenvironment, affecting processes such as EMT and secretion of cytokines to shape the tumor microenvironment [10–12]. In fact, lncRNAs are attractive candidates for cancer biomarkers because of their unique expression patterns and stability. This is represented by PCA3, in the diagnosis and prognosis of prostate cancer, and by HULC, in hepatocellular carcinoma [13, 14].

Especially, the most well-recognized metastasis-associated lung adenocarcinoma transcript 1 (MALAT1) and HOTAIR in cancer biology have become increasingly important in hematological malignancy, including acute myeloid leukemia (AML) [15]. It was initially identified in non-small-cell lung cancer, modulating gene expression, alternative splicing, and nuclear structure. High expression of MALAT1 relates to poor prognosis and recurrence in AML patients [16, 17]. It binds the epigenetic regulatory representative HOTAIR to the PRC2 and LSD1 complexes, leading to the repression of tumor suppressor genes, which contributes to a leukemic transition and chemoresistance in AML [18, 19]. The focus on these lncRNAs in AML points to the use of such transcripts as biomarkers and as therapeutic targets, opening new avenues for personalized medicine treatment of this complex disease [20, 21]. Thus, the main goal of the current review is to focus on the role of LncRNAs, especially of HOTAIR and MALAT1, in diagnosis, prognosis, progression mechanism, and treatment of AML.

## 2. Structure and Function of LncRNAs

### 2.1. LncRNA Structure and Classification

Long noncoding RNAs (lncRNAs) are RNA molecules that play essential regulatory roles within cells. A complex structural organization characterizes lncRNAs, with various forms of structural motifs such as stem-loops, bulges, hairpins, and pseudoknots. Hydrogen bonding and base pairing stabilize these motifs of the lncRNA structure to give characteristic regions of local folding that define individual functional domains. This versatility in structural organization imparts on lncRNAs the capability to function as scaffolds, guides, or decoys to interact with proteins, DNA, and other RNA molecules. The resulting interactions, in turn, modulate a variety of cellular processes, from gene expression to chromatin remodeling, and RNA regulation—all testifying to their importance in being integral components of gene regulation and cellular homeostasis [22].

Accordingly, lncRNAs based on the genomic position related to the protein coding gene and mode of action are classified into several classes such as antisense, sense, intronic, bidirectional, and intergenic [4]. Antisense lncRNAs are those transcribed from the opposite strand. The bidirectional lncRNAs arise similarly from the opposite strand but mostly near the promoter of a coding gene, intronic lncRNAs arise from introns of protein coding genes, while intergenic lncRNAs arise between protein-coding genes [23, 24].

### 2.2. Functional Mechanisms of LncRNAs

LncRNAs are playing roles in a wide range of molecular processes contributing to the hallmarks of cancer. The subsections described below outline mechanisms of lncRNAs in cancer, intermingled with examples to show their influence on cancer progression, metastasis, and resistance to therapy.

1. LncRNAs may act as transcriptional regulators that activate or repress the expression of target genes. Some lncRNAs function as enhancer RNAs, in other words, enhancer RNAs (eRNAs) that directly enhance the nearby gene's transcription. For example, promoter of cyclin-dependent kinase inhibitor 1A (CDKN1A) antisense DNA damage–activated RNA (PANDAR) regulates the expression of CDKN1A, which is an important cell cycle regulator, by binding to its promoter [25]. The regulation of PANDAR itself pertains to cell survival pathways and thus is highly relevant in tumorigenesis and therapeutic resistance in cancers such as lung and breast cancers [1].
2. Epigenetic regulation: LncRNAs function mostly as regulators of the state of chromatin by guiding the chromatin-modifying complexes to particular genomic loci. This would either cause a repression or an activation of transcription. An example is XIST, mediating the inactivation of the female X-chromosome, and functioning as a scaffold to recruit PRC2 toward the inactivation of one X chromosome [26].
  Another example is UCA1 interacting with the PRC2 complex to epigenetically regulate the histone methylation and acetylation patterns. In the case of bladder cancer, UCA1-mediated chromatin modifications promote the silencing of tumor suppressor genes, thus increasing the oncogenic potential of bladder cancer cells [27].
3. Post-transcriptional regulation
  LncRNAs can function similar to miRNA sponges, which will bind the miRNAs and inhibit interaction of these miRNAs with their mRNA targets, a process through which post-transcriptional regulation of gene expression is carried out. One of the well-characterized lncRNAs is H19, functioning as a sponge for miR-675 in regulating oncogenes and tumor suppressors originating from cancers of various types [28].
4. Protein interaction and modulation
  LncRNA transcripts may interact directly with the proteins to influence the stability, activity, or subcellular localization. For instance, nuclear enriched abundant transcript 1 (NEAT1) interacts directly with paraspeckle proteins and influences nuclear organization and gene expression. Poor prognosis due to dysregulated NEAT1 in prostate and breast cancer contributes to aggressive phenotypes through paraspeckle stabilization [29].
  Meanwhile, the protein PVT1 binds to and stabilizes the MYC protein, thus amplifying MYC-driven oncogenic pathways. Overexpression of the PVT1 has been identified in colorectal, breast, and ovarian cancers, where it performs some critical functions concerning proliferation and survival of cancer cells [30].
5. Modulation of RNA splicing
  LncRNAs have been involved in controlling alternative splicing, and one of the consequences is the generation of numerous protein isoforms that may drive cancer. For example, LINC-UBC1 controls the splicing of genes related to cell cycle and apoptosis. In colorectal cancers, its alternative splicing mediated by LINC-UBC1 contributes to survival by generating prosurvival isoforms of important proteins [31].
  Steroid receptor RNA activator (SRA) is another example; in breast cancer, it alters the splicing of the estrogen receptor alpha, which in turn modifies the estrogen signal and stimulates the growth of the tumor [32].
6. Cellular localization and signaling
  LncRNAs realize their functions according to specific subcellular compartments. For instance, lncRNA-p21 expresses its localization to the nucleus, where it regulates the transcription of stress-responsive genes. Others, like MALAT1, localize to nuclear speckles, where pre-mRNA splicing is regulated. Their ability for subcellular distribution to distinctive cellular structures points to crucial functions these lncRNAs play in stress response and metastasis [33].
7. Regulation of stem cell maintenance and differentiation
  LncRNAs play significant roles in regulating the pluripotency and differentiation of stem cells. For instance, TERRA (telomeric repeat-containing RNA) controls telomere length through regulation of telomerase activity, which is crucial to both stem cell activity and tumor cell immortality. TERRA shows abnormal expression in glioblastoma and hematologic malignancies and further promotes uncontrolled cell proliferation (Deng et al., 2009). (H19) is an lncRNA that is highly expressed during embryonic development. It controls the cell differentiation of the stem cells and participates in cancers such as colorectal, probably because of alterations in differentiation pathways that may modulate tumor growth and metastasis. Thus, H19 forms not only an interesting candidate for understanding development but also for disease because of the effects it may have on cell fate control [34].
8. Regulation of apoptosis
  Apoptosis may be induced or suppressed via lncRNAs and thus directly impact cell survival. In hepatocellular carcinoma HCC, lncRNA-ATB has also been shown to promote oncogenesis by interfering with the expression of genes that are pro-apoptotic [12]. Similarly, lncRNA-CCND2 exerts its influence on cell cycle progression and apoptosis via interactions with key regulators of the cell cycle and thus has emerged as a critical determinant of survival in ovarian and lung cancers and chemoresistance [35].

### 2.3. LncRNAs in Cancer

#### 2.3.1. General Mechanisms of LncRNA Roles in Cancer

In cancer, lncRNAs exert an important role in tumorigenesis and the process of cancer progression through modifying several cellular aspects [36]. These noncoding sequences of RNAs interact with chromatin, transcriptional factors, and other regulatory molecules to alter the gene expression profile [1]. Dysregulation in lncRNA expression in cancer alters the appropriate balance between oncogenes and tumor suppressors, which is essential for several cellular processes; thus, this regulates cell proliferation, apoptosis, metastasis, and drug resistance in cancer. Doing so by acting as molecular scaffolds, decoys, or guides for chromatin-modifying complexes, lncRNAs facilitate the activation or repression of genes critical to tumor survival and expansion [37]. They are centrally positioned in maintaining the hallmarks of cancer due to their ability to influence signaling pathways, metabolic regulation, and cell fate decisions, leading to aggressive tumor behavior and poor clinical outcomes. A number of lncRNAs have been identified to drive specific cancer-related mechanisms; they have, thus, been considered integral in the understanding and targeting of malignancies [38].

The role of lncRNAs in cancer can be summarized in the following hallmarks of cancer:

##### 2.3.1.1. Sustaining Proliferative Signaling

LncRNAs regulate oncogene expression and activity and further perpetuate proliferative signaling. The PVT1 lncRNA interacts with MYC to stabilize the protein product from MYC and prevent its degradation. This strengthens MYC-driven transcriptional activation and proliferation of cancer cells. Consequently, there is an increased expression of genes involved in cell growth and cell division, which in turn enhance tumor progression [30]. CCAT2 binds to the promoter region of MYC to increase its expression through facilitating transcription machinery assembly, leading to the upregulation of genes driving colorectal cancer cell growth [39].

##### 2.3.1.2. Evading Growth Suppressors

LncRNAs can repress tumor suppressor genes. Thereby, lncRNAs allow the cancer cells to escape from growth control [36]. ANRIL epigenetically represses the INK4b-ARF-INK4a tumor suppressor locus through interaction with the PRC2 complex, resulting in histone H3K27 trimethylation and gene silencing, promoting proliferation of tumor cells [40]. ANRIL is associated with numerous types of cancers, especially prostate and breast cancer [41, 42]. The lncRNA UCA1 suppresses the expression of tumor suppressors through the direct interaction with chromatin-modifying complexes like PRC2, which alters the target gene expression responsible for cell cycle regulation and apoptosis [43].

##### 2.3.1.3. Resisting Cell Death

LncRNAs modulate the pathways of apoptosis for survival advantages of the cancerous cell. The lncRNA-ATB operative in hepatocellular carcinoma suppresses apoptosis by downregulation of pro-apoptotic factors and upregulation of anti-apoptotic proteins. It supports epithelial-to-mesenchymal transition, thus facilitating metastasis as well [44]. Long noncoding RNA SNHG1 stabilizes anti-apoptotic proteins in gastric cancer. This accordingly modulates genes such as Bcl-2 and Bax, hence making cells more resistant to apoptotic signals [45].

##### 2.3.1.4. Enabling Replicative Immortality

Long noncoding RNA SNHG1 has the ability to stabilize anti-apoptotic proteins, while modulating genes like Bcl-2 and Bax, making cells resistant to apoptotic signals in gastric cancer. [46]. H19 also interacts with the telomerase complex, hence contributing to the telomerase activity regulating telomere length and thereby causing extended life span of the cancerous cells [47].

##### 2.3.1.5. Inducing Angiogenesis

It has been suggested that lncRNAs encourage angiogenesis. LINC-PINT promotes tumor vascularization via regulating the production of VEGF and other angiogenic genes [48]. LncRNA-CCAL mediates VEGF expression that affects the function of endothelial cells, promoting neovascularization within the tumor and hence enhancing its growth and progression in colorectal cancer [49].

##### 2.3.1.6. Activating Invasion and Metastasis

LncRNAs promote the metastasis and invasive capabilities of tumor cells. LncRNA-ATB exerts its function by inducing EMT, which enhances invasion process of tumor cells, hence leading to metastasis in various types of cancer including liver cancer (HCC) [50]. LncRNA-MIAT contributes to metastasis by influencing pathways related to cell adhesion and migration, which involves modulating proteins concerned with cell-to-cell adhesion as well as interactions with the extracellular matrix to enhance invasive capability [51].

##### 2.3.1.7. Cellular Metabolism Deregulation

LncRNAs have the capability to change the cancerous cell metabolism for facilitating high tumor energy requirements. Long noncoding RNA UCID regulates glucose metabolism by controlling enzymatic steps of glycolysis and glucose uptake, hence promoting the survival of cancerous cells under metabolic stress circumstances [52]. LncRNA PU.1 AS influences metabolic processes like glycolysis and oxidative phosphorylation and controls the metabolism of energy along with tumor growth [53].

#### 2.3.2. LncRNAs Act as Tumor Suppressors and Oncogenes

By virtue of their involvement in cellular processes, lncRNAs can act either as oncogenes or as tumor suppressors. The oncogenic lncRNAs, often referred to as “onco-lncRNAs,” facilitate tumorigenesis through promotion of cell proliferation, invasion, and metastasis and inhibition of cell death [54]. For example, overexpression of HOTAIR suggested to be associated with several cancers, such as breast, colorectal, and hepatocellular carcinomas, in which the expressed HOTAIR promotes tumorigenesis by reprogramming chromatin states and silencing tumor suppressor genes through H3K27me3 [55].

Some lncRNAs can play the role of tumor suppressors, too. A major example is represented by MEG3, whose expression has been reported to be downregulated in several subtypes of cancer, such as gliomas and hepatocellular carcinoma [56]. MEG3 performs this role through interaction with p53, whereby it enhances tumor-suppressive activity, as well as through modulation of miRNA and other regulatory molecules involved in cell cycle arrest and apoptosis. Loss of MEG3 expression through either genetic or epigenetic alterations results in tumorigenesis via disturbance of such regulatory networks [57].

### 2.4. MALAT1 and Its Role in Cancer

**Figure 1:**
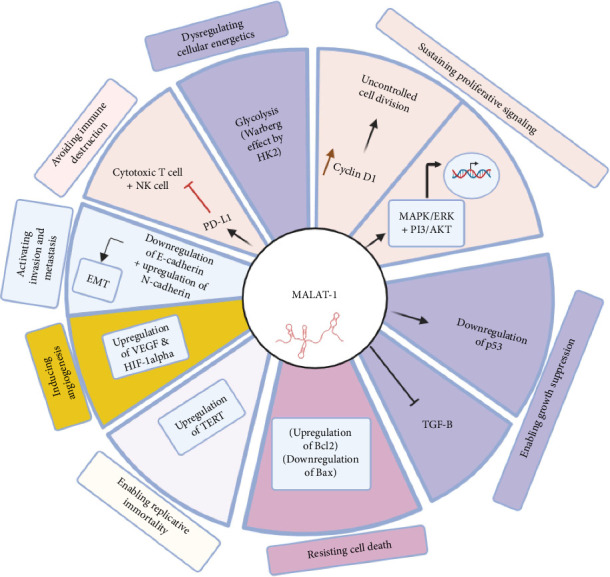
Functional roles of the long noncoding RNA MALAT1 in cancer progression and hallmark pathways. This figure shows the multifaceted role of lncRNA MALAT1 in driving the acquisition of several hallmarks of cancer. MALAT1 has critical functions that promote sustained proliferative signaling via induction of Cyclin D1 and activation of MAPK/ERK and PI3K/Akt pathways while enabling growth suppression through repression of the P53 gene, dampening TGF pathways. MALAT1 also confers resistance to cell death by upregulating the antiapoptotic BCl2 gene while downregulating the proapoptotic Bax gene. It confers replicative immortality through upregulation of telomerase expression-TERT. Furthermore, MALAT1 promotes angiogenesis through overexpression of VEGF and HIF-1α, invasion, and metastasis via cadherin expression modulation, and immune destruction is evaded by interference with T cytotoxic and NK cell functions. Finally, MALAT1 dysregulates cellular energetics by inducing the Warburg effect, which favors aerobic glycolysis in cancer cells. All these pathways taken together underpin the immense role of MALAT1 in cancer pathogenesis.

MALAT1 is a long noncoding RNA (lncRNA) recognized for its link to cancer metastasis, especially in certain types of lung cancer (Such as non-small-cell lung cancer) [58]. It was discovered in 2003 during research focused on genes associated with lung cancer prognosis. It was proposed to be significantly upregulated in metastatic tumor cells. [16]. This discovery led to interest in MALAT1's potential role in cancer development. Early research connected high MALAT1 expression with enhanced cell migration, invasion, and poorer cancer outcomes, establishing it as an important factor in cancer metastasis (Figure 1) [59]. It has emerged as a critical player in cancer biology, with influences on almost every imaginable aspect of disease progression and management—from diagnosis and prognosis to drug responses and therapeutic outcome [60].

MALAT1, therefore, has great potential as a diagnostic biomarker. The detection of high levels of MALAT1 in plasma or tissue samples most often indicates the presence of cancer. For example, in NSCLC, high expression of MALAT1 is associated with malignancy; hence, it can be used as a noninvasive diagnostic biomarker [61]. Similarly, the expression level of MALAT1 in prostate cancer can indicate malignant versus benign conditions, improving diagnostic accuracy when used in combination with conventional imaging and biopsy techniques [62].

Aside from diagnosis, MALAT1 also functions as a prognostic marker; thus, its overexpression normally accompanies unfavorable clinical features such as the presence of disease at an advanced stage, or overall lower survival rates [63]. MALAT1 in breast cancer maintains a close relationship with aggressive features of tumors and a poor prognosis [64]. In this regard, MALAT1 expression can predict recurrence of the disease and may provide important information about post-treatment follow-up and follow-up strategy [65].

Its involvement in drug responses underlines its contribution to therapeutic efficacy. MALAT1 has been implicated in a drug-resistant mechanism across a wide range of cancers. MALAT1, for example, has been shown to mediate resistance against paclitaxel in ovarian cancer through the modulation of mechanisms related to drug transportation and apoptosis, hence affecting its efficacy [66]. Similarly, MALAT1 modulates the sensitivity of tumor cells to tyrosine kinase inhibitors in NSCLC and, by extension, treatment outcome. This outlines its importance for personalized treatment approaches [67].

MALAT1 has also therapeutically become a target for cancer therapy. The development of approaches targeting the inhibition of MALAT1 expression or functions are under development against its tumor-promoting activities. Other investigative approaches which can diminish the expression of MALAT1 include small molecules, antisense oligonucleotides, or RNA interference [68]. These may increase additional treatments and overcome resistance mechanisms. Future combination of MALAT1 inhibitors with standard chemotherapy or targeted therapy may also be explored for further improvement in therapeutic outcomes [69].

MALAT1 promotes tumor progression in colon cancer by regulating the expression of genes responsible for the control of the cell cycle, which include cyclins and cyclin-dependent kinases. Such a regulation might thus contribute to uncontrolled cell division of tumor cells—the hallmark of cancer [70].

With regard to metastasis, MALAT1 promotes the invasive and disseminating capabilities of tumor cells. It induces EMT and modulates the expression of matrix metalloproteinases (MMPs) necessary in tissue invasion. For instance, MALAT1 induces EMT in breast cancer, enhancing the invasive capability of tumor cells and metastatic dissemination [70]. MALAT1 also regulates the signaling pathways concerned with metastasis, such as the TGF-β and Wnt/β-catenin pathways, for further promotion of tumor spread [71].

It also dramatically modulates cell survival and apoptosis. MALAT1 exerts an anti-apoptotic effect since it controls the expression of both anti-apoptotic and pro-apoptotic proteins. In gastric cancer, MALAT1 modulates the Bcl-2/Bax ratio toward promoting survival and death resistance of cancer cells [72]. MALAT1 also promotes adaptation of tumor cells to stressful conditions of hypoxia and nutrient deprivation commonly occurring in the tumor microenvironment [73].

### 2.5. HOTAIR in Cancer

**Figure 2:**
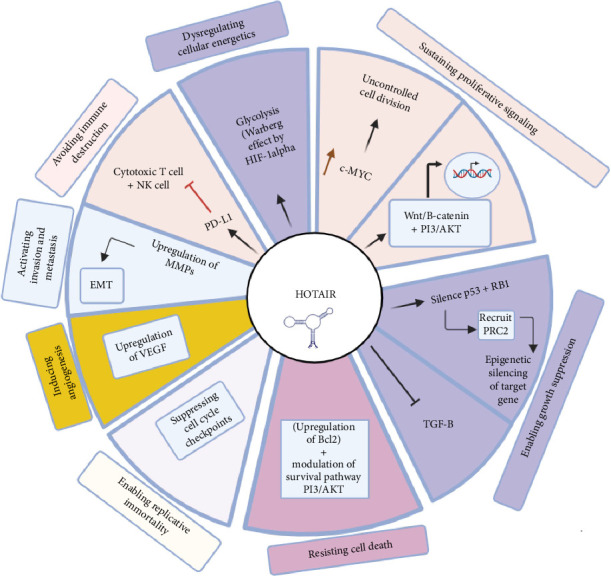
The functional role of the lncRNA HOTAIR in cancer progression and hallmark pathways. This figure depicts the multifaceted roles of HOTAIR lncRNA in facilitating cancer hallmarks. LncRNA HOTAIR maintains proliferating signaling through induction of c-MYC, activation of the Wnt/β-catenin pathway, and activation of the PI3K/Akt pathway. The growth suppressors were enabled by lncRNA HOTAIR through silencing P53 and RB1 genes and inhibiting TGF pathways. HOTAIR resists cell death by upregulating the antiapoptotic gene BCl2 and also modulates survival via the PI3K/Akt pathway. Replicative immortality is further sustained through suppression of cell cycle checkpoints by HOTAIR. It also enhances angiogenesis by overexpression of VEGF, aids invasion and metastasis through the upregulation of matrix metalloproteinases, and avoids immune destruction by interfering with cytotoxic T-cells and NK cell functions. Finally, HOTAIR dysregulates cellular energetics by inducing the Warburg effect through enhancement of the HIF-1 signaling pathway and, in this way, promotes aerobic glycolysis in tumor cells. In summary, these pathways underpin the critical contributions of HOTAIR in cancer pathogenesis.

HOTAIR is a genre of lncRNA that has gained extraordinary interest because of its comprehensive involvement in almost all aspects of cancer, including influencing diagnosis and prognosis to drug responses, cancer progression and metastasis, and cell survival (Figure 2). Most often, the levels of HOTAIR expression are considerably upregulated in tumor tissues than in neighboring noncancerous tissues. For example, in the case of breast cancer, high expression of HOTAIR has been associated with malignant transformation and can thus be used as an early diagnostic marker of the disease itself [74]. Meanwhile, in HCC, high expression of HOTAIR is associated with the presence of cancer. It therefore makes HOTAIR a noninvasive diagnostic tool when its levels are measured in blood or urine samples [75].

Clinical outcomes have also been correlated with the expression of HOTAIR in a reasonably coherent manner across different cancers. The high expression of HOTAIR has been associated with advanced disease stages, aggressive tumor behavior, and reduced overall survival. For example, in gastric cancer cases, high levels of HOTAIR indicate a bad prognosis and are associated with a higher risk of disease progression and shorter survival of the patients [76]. It is also reflected in the high expression of HOTAIR in colorectal cancer, related to the progression and metastasis of tumors and hence a marker for prognosis [77].

A wide range of chemotherapy drugs were found to alter sensitivity to HOTAIR in tumor cells. For example, in ovarian cancer, HOTAIR mediated resistance to cisplatin through modulation of pathways in drug metabolism and apoptosis that affected treatment outcome [78]. Moreover, the involvement of HOTAIR in drug resistance mechanisms makes the expression level of this lncRNA relevant for designing personalized protocols of treatment [79].

Thus, from a therapeutic point of view, targeting HOTAIR may represent one of the useful strategies for the treatment of cancer. Other strategies currently under investigation for silencing its expression or action include RNA interference, antisense oligonucleotides, and small molecule inhibitors. For instance, small molecules interfere with the interaction of HOTAIR with chromatin-modifying complexes, an approach that has been pursued in preclinical treatments [80]. Combination treatments targeting HOTAIR with conventional chemotherapy or targeted therapies may eventually result in an overall increase in efficacy of therapy and bypass some mechanisms of resistance [81].

The role of HOTAIR in carcinogenesis is enormous. Many of the leading processes concerning tumor development and growth were subjected to modulation by HOTAIR. In the case of breast cancer, HOTAIR controls genes responsible for cell proliferation and survival through interaction with polycomb repressive complexes, hence enhancing tumor growth and promoting its progression [82]. This has also been demonstrated in the case of pancreatic cancer, where HOTAIR modulates the cell cycle and apoptosis, thus imparting an aggressive nature to the tumor [83].

Another important aspect of metastasis refers to the ways in which HOTAIR exerts critical effects. Indeed, HOTAIR enhances invasive and migratory properties of tumor cells by regulating EMT and extracellular matrix remodeling. As a specific example, in lung cancer, HOTAIR enhances cell migratory and invasive capabilities through the regulation of the expression levels of some EMT markers, including MMPs, that contribute to metastatic dissemination [84].

HOTAIR regulates cell survival and apoptosis. It interferes with the balance between pro-apoptotic and anti-apoptotic signals, influencing resilience of malignant cells. In this regard, in endometrial cancer, HOTAIR exerts anti-apoptotic activity by modulation of expression of apoptosis modulators including Bcl-2 and Bax, resulting in increased survival of malignant cells and their resistance to cell death [85].

## 3. LncRNAs in AML

### 3.1. Cellular and Molecular Basis of AML: An Overview

The landscape of genetic and molecular features in AML is just too complicated; it predisposes due to a wide array of mutations and chromosomal abnormalities that can drive the initiation and progression of the disease. Some of the key mutations in AML include, among others, NPM1, IDH2, and p53 mutations, each having its own unique contribution to the pathogenesis of AML [86].

Among genetic alterations, NPM1 mutations are the most common in AML and generally relate to a good prognosis when presented alone [87]. The protein NPM1 has roles assigned in the nucleolar structure and function; its mutations often cause aberrant cytoplasmic localization. Such a mislocalization disrupts its normal role in ribosome biogenesis and cellular homeostasis, promoting leukemogenesis [88]. NPM1 mutations often meant peculiar clinical and molecular features, such as high blast counts and specific gene expression signatures [89].

The p53 tumor suppressor gene guards genomic integrity and cell cycle regulation and contains frequent mutations in AML. Such mutations compromise the induction of apoptosis and cell cycle arrest by p53 in response to DNA damage. Loss-of-function p53 enhances genomic instability and drives AML forward. Indeed, p53 mutational status is a critical factor in therapy response and prognosis in AML patients [90]. In AML, the bone marrow microenvironment represents the hallmark of disease course and therapeutic resistance. The interaction of leukemia cells with the surrounding stromal components, especially the mesenchymal stem cells, extracellular matrix, and various cytokines, has a great bearing on the disease dynamics [91].

These leukemia cells interact with the bone marrow stroma and create a supportive niche that enhances leukemia cell survival and proliferation. This is an interaction that involves adhesion molecules and cytokines, which facilitate leukemia cell homing and retention within the marrow [92]. For example, the interaction of AML cells with bone marrow extracellular matrix, mediated by integrins among other adhesion molecules, facilitates the maintenance of the leukemic cell population and confers resistance to therapy [93].

The bone marrow microenvironment synthesizes several cytokines and growth factors that may play a role in the pathology of AML. For instance, IL-6 and other inflammatory cytokines-secreting bone marrow stromal cells support the growth and survival of the leukemia cells, thereby setting up an inflammatory milieu that contributes to the progression of disease [94]. More importantly, the bone marrow microenvironment can affect response to therapy by promoting chemotherapy and targeted therapy resistance through certain cytokines and growth factors [95].

### 3.2. Role of LncRNA in AML Progression

LncRNAs represent significant players in the process of AML progression, given their involvement in controlling molecular processes such as cell proliferation, differentiation, apoptosis, and chemoresistance [96]. In fact, these noncoding RNAs tune the expression levels of genes, from chromatin-remodeling and transcriptional control to post-transcriptional processing, thereby contributing to a great extent to the aberrant cellular behaviors of AML [97]. Increasingly, studies uncover the multifaceted ways in which lncRNAs support AML development through various mechanisms and are crucial key regulators in leukemogenesis and hence a potential target for therapy.

A characteristic feature of AML is abnormal expression of lncRNAs. The deregulated lncRNAs themselves have crucial roles as oncogenes or tumor suppressors in the regulation of leukemic cell functions [98]. Oncogenic lncRNAs promote proliferation and inhibit cell differentiation, while tumor-suppressive lncRNAs are silenced to further support the development of AML. Overexpression of other lncRNAs, including LINC00152, NEAT1, and PVT1, similarly activates oncogenic pathways or suppresses tumor suppressors in AML. For example, LINC00152 has been reported to promote leukemic proliferation by modulating transcription factors and interaction with regulators of the cell cycle, thus enabling uncontrolled cell division [99].

These tumor-suppressor lncRNAs are normally downregulated in AML, leading to fewer inhibitory signals critical for the control of cell proliferation and enhancement of apoptosis. For instance, MEG3 triggers the p53 pathway, inducing cell cycle arrest and the apoptosis of AML cells. Loss of this mechanism due to loss of expression of MEG3 allows leukemic blasts to grow unchecked [100].

LncRNAs facilitate unchecked proliferation of AML cells by regulating critical pathways for the control of the cell cycle and growth signaling. Most oncogenic lncRNAs mediate their effects by upregulating the expression or activity of transcription factors and signaling proteins that favor cell division. For example, LINC00152 upregulates the key transcription factor MYC, which trans-activates genes responsible for forward progression during the cell cycle, thus maintaining unblocked cell division in AML cells [101]. Interaction with chromatin modifiers by LINC00152 drives the transcriptional activation of genes related to the cell cycle, including those coding for CDKs, which were responsible for high-speed leukemic cell proliferation [102].

One of the hallmarks of AML is the failure of myeloid progenitors to differentiate into mature blood cells due to the accumulation of immature leukemic blasts [103]. LncRNAs are significantly involved in this differentiation block through the activity of key transcriptional regulators. For instance, LINC00470 represses the function of PU.1 and CEBPα, two critical transcription factors for myeloid differentiation, by directly occupying the promoter regions of these genes. It represses the factors necessary for the purpose and, in turn, keeps the AML cells in an undifferentiated and proliferative state that helps the disease progress [104]. LncRNAs also affect apoptosis, an important process that eliminates damaged or otherwise abnormal cells, including leukemic blasts. Oncogenic lncRNAs promote persistence and disease development through the inhibition of apoptosis, which enables the survival and accumulation of leukemic cells. Among the critical drivers of inhibited apoptosis in AML is NEAT1. This alters the expression of the anti-apoptotic protein BCL-2, which prevents planned cell death and permits AML cells to proliferate without going through apoptosis [105].

### 3.3. Role of MALAT1 in AML

#### 3.3.1. Expression Patterns and Clinical Relevance in AML

Many cancers are studied for MALAT1 expression, and among them, its involvement in AML holds a great place. The expression of MALAT1 in AML is much higher compared to the normal hematopoietic cells, and quite frequently, patients with AML have its upregulation. Moreover, according to several studies, high expression levels of MALAT1 relate to poor clinical outcomes, such as a shorter overall survival rate and disease-free survival [106]. Overexpression of MALAT1 usually points to aggravation in the features of the disease, that is, higher counts of blasts and a higher recurrence rate. MALAT1 clinical value in AML is underlined by its potential use as a prognostic biomarker. High MALAT1 expression independently predicts poor prognosis and is related to adverse risk features in AML patients. The prognostic value of MALAT1 is supported by the consistent association with unfavorable disease characteristics and resistance to conventional treatments [107].

#### 3.3.2. Influence on AML Cell Proliferation, Survival, and Migration

MALAT1 regulates AML proliferation, survival, and migration—each representing important pathways in the disease progression of leukemia. MALAT1 executes its function at least partly through the regulation of dynamics in cell cycle progression. MALAT1 facilitates AML cell division by enhancing the G1-phase to S-phase transition in the cell cycle. This process is mediated through interactions with key players in regulators of the cell cycle, including various types of cyclins and different cyclin-dependent kinases [108]. In addition to promoting proliferation, MALAT1 has been implicated in survival mechanisms of AML cells. It achieves this by regulation of apoptosis pathways of cell death. MALAT1 interfered with the apoptotic machinery by altering anti-apoptotic protein expression and inhibiting pro-apoptotic factors. For instance, MALAT1 has been shown to upregulate the anti-apoptotic protein Bcl-2 while downregulating the pro-apoptotic factor Bax, leading to the promotion of cell survival and resistance to therapy [109].

**Figure 3:**
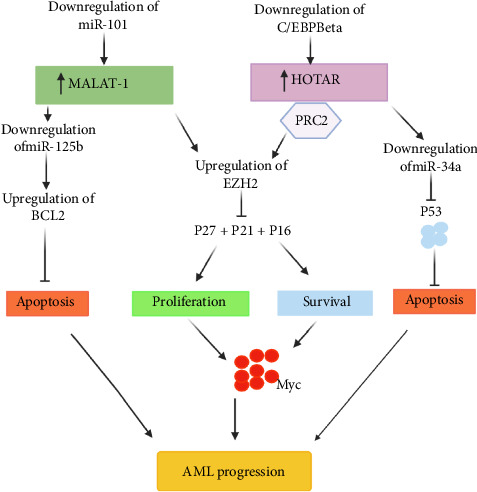
HOTAIR and MALAT1 mechanisms in the progression of acute myeloid leukemia. The proposed mechanisms of lncRNAs MALAT1 and HOTAIR contributing to AML progression are schematically represented here. MALAT1 sponges miR-101 and miR-125b, which stabilizes EZH2 through repression of its mRNA degradation, further epigenetically silencing cell cycle inhibitors p21, p16, and p27, therefore promoting unlimited cell proliferation and increasing BCL2 expression that contributes to cell survival. HOTAIR acts through interaction with PRC2 to recruit EZH2, leading to tumor suppressor silencing, which includes p53, p21, p16, and p27. Besides, downregulation of CEBPβ and sequestration of miR-34a also facilitate AML cell proliferation and resistance to apoptosis. These collective mechanisms define how MALAT1 and HOTAIR contribute to AML pathogenesis.

MALAT1 is one of the prominent lncRNAs that influences cancer cell behavior mainly by acting as competing endogenous RNA (ceRNA), especially through interactions with microRNAs like miR-101 and miR-125b. MALAT1 binds and sequesters miR-101 and miR-125b, thus preventing them from executing their repressive function on their downstream targets. Generally, miR-101 and miR-125b function as tumor suppressors. They target the critical oncogenes and other regulatory molecules involved in pathways related to cell proliferation, apoptosis, and metastasis [106, 110]. In this regard, MALAT1 binding to these miRNAs indirectly facilitates stability and expression of the target genes, including but not limited to EZH2, a histone methyltransferase involved in chromatin remodeling and transcriptional repression [111]. MALAT1 promotes epigenetic changes that support the survival, proliferation, and metastasis of cancer by modulating the expression of EZH2 through its interaction with miRNA (Figure 3).

Besides upregulation of EZH2, MALAT1 controls factors associated with apoptosis regulation, including p21, p16, p27, and BCL2. The stabilized EZH2 due to MALAT1-mediated miRNA sponging epigenetically represses tumor suppressors like p21, p16, and p27. These genes are essential CDKIs that are required for cell cycle arrest and apoptosis. The downregulation of these CDKIs allows the unhindered proliferation of cells and thereby enables tumor cells to bypass cell cycle control [100, 112, 113]. Moreover, MALAT1 may promote the expression of BCL2, an anti-apoptotic protein, which further enhances resistance to programmed cell death in cancer cells. By downregulating pro-apoptotic factors and upregulating the amount of BCL2, MALAT1 maintains cellular survival signals within the tumor microenvironment conducive to a more aggressive cancer phenotype [106].

The other important aspects of the role of MALAT1 in leukemia concern its modulation of migration and invasion in AML cells. The MALAT1 noncoding RNA exerts an effect on the EMT process and further on the expression of MMPs; this promotes the process of cell migration and invasion in the case of leukemia. MALAT1 overexpression significantly increased the invasive capacity of AML cells by upregulating MMP-2 and MMP-9 expressions, hence increasing the degradation of proteins of the extracellular matrix, promoting metastasis [111].

#### 3.3.3. Interaction With Key Signaling Pathways in AML

MALAT1 regulates several key signaling pathways that are implicated in the pathogenesis of AML. Of these, one of the key pathways targeted by MALAT1 has been identified to be the PI3K/Akt pathway. Till now, MALAT1 has been demonstrated to activate this pathway and thus facilitate the survival and proliferation of cells at an increasing rate. It also interacts with PI3K and Akt, promoting their activation to subsequent downstream events that result in growth in leukemia cells [112]. Another important pathway of MALAT1 action is the Wnt/β-catenin pathway. MALAT1 mediates its role through interaction with β-catenin and regulation of its nuclear translocation, thus modulating the Wnt/β-catenin pathway. This results in the upregulation of target genes, c-Myc and cyclin D1, which play critical roles in cell proliferation and survival. These findings thus indicate the role of MALAT1 in maintaining leukemic phenotype and disease progression [113].

MALAT1 further modulates the NF-κB signaling pathway, one of the major factors in the inflammation and immune response processes. MALAT1 activates NF-κB by interacting with its regulatory complex to further facilitate the nuclear localization of NF-κB. The MALAT1-induced NF-κB promoted expression of pro-inflammatory cytokines and growth factors, thus promoting survival and proliferation in AML cells [114]. Another interesting aspect is the expression of MALAT1 in the Notch signaling pathway. MALAT1 modulates the transcriptional activity of the Notch receptor and its target genes, thus maintaining the Notch signaling pathway in AML cells. This has been one of the leukemic stem-cell-like states associated with resistance to therapy and disease relapse [115].

### 3.4. Role of HOTAIR in AML

#### 3.4.1. Expression Patterns and Clinical Relevance in AML

A lncRNA called HOTAIR is known to have important regulatory roles in a variety of malignancies, including AML. Normally, it is overexpressed in AML compared to normal hematopoietic cells. The expression level has been associated with disease severity and prognosis. High-expression HOTAIR relates to adverse clinical outcomes, as shown by overall survival and progression-free survival [116]. Overall, high expression of HOTAIR is indicative of a more aggressive leukemia phenotype, usually presenting with higher blast counts and predisposition to relapse [117].

This has been further underlined by the potential of HOTAIR serving as a prognostic biomarker in AML. Indeed, several studies documented that a level of HOTAIR expression could independently predict poor prognosis in AML patients. High levels of HOTAIR are associated with adverse characteristics of disease, poor response to conventional therapies, and increased risk of treatment failure [116].

#### 3.4.2. Influence on Epigenetic Regulation and AML Progression

HOTAIR epigenetically regulates genes central to the pathogenesis of AML. Its primary mechanism involves acting as a molecular scaffold that binds to Polycomb Repressive Complex 2 (PRC2) at its 5′ domain and LSD1 (lysine-specific demethylase 1) at its 3′ domain. This dual interaction facilitates coordinated chromatin modification by promoting H3K27 trimethylation and H3K4 demethylation, respectively, at target loci. The resulting repressive histone marks lead to silencing of tumor suppressor genes and the activation of leukemogenic programs, thus contributing to AML development and progression [118, 119].

Additionally, HOTAIR has been shown to influence DNA methylation patterns through modulation of DNA methyltransferase activity, promoting hypermethylation of tumor suppressor gene promoters and reinforcing transcriptional silencing [120, 121].

Beyond these effects, HOTAIR exemplifies how lncRNA structure directly influences function—its modular domains enable simultaneous recruitment of distinct chromatin-modifying complexes, enhancing its ability to reshape the AML epigenome [9].

In contrast, other lncRNAs like MALAT1 contribute more through nuclear structural regulation than through direct epigenetic remodeling. MALAT1 is enriched in nuclear speckles where it modulates pre-mRNA splicing, mRNA stability, and post-transcriptional gene regulation by interacting with splicing factors such as SRSF1. Although not a classic epigenetic regulator, MALAT1 affects gene expression programs essential to leukemic cell survival and may indirectly influence the epigenetic state through transcriptomic reprogramming [2].

Collectively, these findings underscore that lncRNAs contribute to AML progression not only through epigenetic repression or activation of gene expression but also via 3D chromatin remodeling and nuclear structural organization. Their distinct mechanisms—whether scaffold-mediated silencing (HOTAIR), nuclear structural modulation (MALAT1), or chromatin topology organization (HOTTIP, HOXBLINC)—highlight the mechanistic diversity and clinical significance of lncRNAs in the leukemic epigenetic landscape [122, 123].

#### 3.4.3. Interaction With Key Signaling Pathways in AML

HOTAIR modulates many important signaling pathways involved in pathogenesis in AML and further elaborates its role in the biology behind leukemia. Amongst these, one is the Wnt/β-catenin pathway, which has been altered by HOTAIR. It interacts with β-catenin and affects its nuclear translocation. Further, the Wnt/β-catenin pathway activated by HOTAIR upregulates its target genes, which promote proliferation and inhibit cell differentiation to develop AML [124].

HOTAIR usually downregulates the expression of the transcription factor CEBPβ, which generally acts as a tumor suppressor by inducing cell differentiation and repressing proliferation. The suppression of CEBPβ mediated by HOTAIR acts to promote differentiation and support a more aggressive phenotype in cancer cells [125]. Another target for direct binding by HOTAIR is miR-34a, a microRNA possessing potent tumor-suppressive functions targeting genes with roles in cell cycle control and apoptosis. HOTAIR can sequester miR-34a from performing its functions [126]. Another target for direct binding by HOTAIR is miR-34a, a microRNA possessing potent tumor-suppressive functions targeting genes with roles in cell cycle control and apoptosis. HOTAIR can sequester miR-34a from performing its functions [127]. One of the core features of its oncogenic role is its influence on EZH2, an essential constituent of Polycomb Repressive Complex 2. HOTAIR binds directly to PRC2, allowing EZH2 to perform the trimethylation of histone H3 on lysine 27, H3K27me3, which in turn causes the transcriptional repression of tumor suppressor genes like p53, p21, p16, and p27 [128]. p53 mediates critical roles in the induction of apoptosis and cell cycle arrest, while p21, p16, and p27 are inhibitors of cyclin-dependent kinases, enhancing tumor-suppressive cell cycle checkpoints that prevent unchecked cellular growth. Activation of these tumor suppressor genes by HOTAIR through direct EZH2-mediated repression allows cancer cells to bypass apoptosis and keep proliferating [129, 130]. This repression enables bypass mechanisms against cell cycle control and apoptosis, promoting tumor progression and resistance to therapy from various kinds of cancers. These interactions, in turn, allow HOTAIR to drive a strong oncogenic epigenetic environment that is promoting malignancy (Figure 3).

HOTAIR also interacts with the Notch pathway, which might have been expected to play a crucial role in maintaining leukemia stem cells by determining cell fate. Indeed, the lncRNA HOTAIR epigenetically regulates the expression of both Notch receptors and ligands, and alters associated downstream signaling events required for AML differentiation and self-renewal [131]. The influence of HOTAIR on Notch signaling perhaps best epitomizes its role in sustaining AML in a leukemic stem cell-like state with the view of promoting disease progress [132].

Moreover, it influences the MAPK/ERK pathway, which also has cell growth and survival functions. The interaction of HOTAIR with the components of the MAPK/ERK pathway increases the activation of ERK1/2 and then enhances target genes in cell proliferation and survival. All this further supports the role of HOTAIR in promoting AML cell growth and resistance to therapy [133].

## 4. LncRNAs and the Bone Marrow Microenvironment in Cancer

In this regard, lncRNAs have increasingly been identified to play critical roles in the regulation of the bone marrow microenvironment, which is crucial for the progress, metastasis, and development of chemoresistance in cancers. These lncRNAs, longer than 200 nucleotides, regulate gene expression, chromatin modification, and protein interaction, thereby influencing the complex interactions between the cancer cells and the BMM. Some of them were implicated in the dysregulation of various cancers, including breast cancer, multiple myeloma, and leukemia, in which they modify major cellular processes such as communication, immune evasion, ECM remodeling, and resistance to therapy that support tumor growth and metastasis [28].

One of the most significant mechanisms of how lncRNAs affect BMM is modulating cellular interplay between tumor cells and other members of the microenvironment such as MSCs, immune cells, and endothelial cells. For instance, lncRNA H19 was identified recently to interfere in breast cancer with the CXCL12/CXCR4 signaling axis—a pathway of paramount importance for the migration and homing of tumor cells toward the bone marrow. This dysregulation favored retention and colonization of cancer cells within the marrow niche, in this way favoring metastatic progression [51]. In the context of multiple myeloma, this has been demonstrated for MIR17HG, which controls the CXCR4 receptor, interacting through BMM and driving survival and proliferation of the tumor cells [134]. For example, in the case of AML, some lncRNAs, like MEG3, are downregulated even in the bone marrow stromal cells themselves and further contribute to creating a pro-tumorigenic microenvironment, promoting the survival of the leukemic cells [28].

Other key functions of lncRNA include their role in modulating angiogenesis for survival and proliferation of the cancerous cells in BMM. Angiogenesis is the development of new vessels independent of the primary tumors and metastatic sites to meet the nutritional and oxygen supply [135]. The long noncoding RNA HOTAIR facilitates angiogenesis through upregulation of vascular endothelial growth factor, hence promoting expansion of blood vessels in the bone marrow to support the survival of metastatic breast cancer cells [39]. Similarly, the lncRNA PVT1 promotes angiogenesis by maintaining stable proangiogenic factors, such as VEGF; hence, cancer cells within the marrow can thrive and establish bone metastases [136].

Further to this role in modulation of communication and angiogenesis, the lncRNAs also take part in remodeling the structure of the extracellular matrix that is highly needed for invasion and migration of cancer cells into the bone marrow. For example, in breast cancer, ANCR represses the expression of MMP-9, an enzyme that degrades ECM, which further promotes the invasion of cancer cells into the bone marrow [51]. Meanwhile, NEAT1, in multiple myeloma, is engaged in upregulation processes involved in the breakdown of ECM by the action of MMPs, enabling malignant cells to invade the bone marrow niche. This means that lncRNAs, upon modulation of the ECM remodeling, significantly contribute to metastatic spread of tumor cells into the bone marrow where they can be protected and find a good environment for further growth [137].

Moreover, lncRNAs participate in the immunosuppressive nature of BMM by allowing tumor cells to have immune-escape capabilities. For example, in the context of breast cancer, lncRNA SNHG1 upregulates the immune checkpoint protein PD-L1, well known to suppress T-cell activities, thereby enabling the tumor cells to evade immune surveillance [138]. This mechanism of immune evasion further extends to prostate cancer, where the lncRNA H19 influences TAM behavior toward creating an immunosuppressive microenvironment and supports tumor growth and metastasis. These mechanisms allow lncRNAs to enable cancer cells to bypass the immune response and to continuously be present within the bone marrow in a protected way [139].

Last but not least, lncRNAs serve as facilitators of chemoresistance. Usually, the bone marrow acts as a kind of sanctuary where the disseminated tumor cells can easily get away from cytotoxic chemotherapy; lncRNAs also contribute to such kinds of resistance [140]. For example, in breast cancer, the long noncoding RNA GAS5 regulates drug efflux transporters such as ABCG2, which actively pumps chemotherapeutic agents out from the tumorous cells, rendering them resistant against such therapies. LncRNA TUG1 in multiple myeloma drove the activation of survival-promoting signaling pathways, including the PI3K/AKT pathway, which conferred resistance against the cytotoxic effects of chemotherapy on the tumorous cells. These lncRNAs confer chemoresistance and ensure that the cancer cells present within BMMs evade therapy to lead to relapse and disease progression [141].

### 4.1. MALAT1 and HOTAIR Action in the Bone Marrow Microenvironment in AML

**Figure 4:**
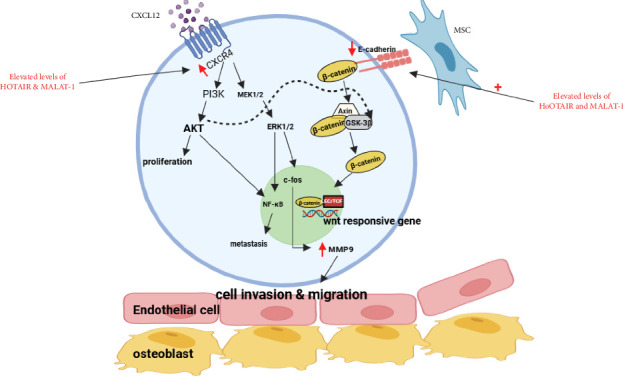
A schematic diagram representing the interaction of MALAT1 and HOTAIR with components of the bone marrow microenvironment: CXCR4, MMP9, and cadherin. This figure depicts the consequences of highly expressed MALAT1 and HOTAIR lncRNAs in AML. The overexpression of MALAT1 significantly stimulates the PI3K/AKT pathway, cell proliferating activity, and decreases apoptosis with a view to maintaining LSCs within the bone marrow niche. It also enhances CXCR4 and its ligand CXCL12, which facilitates the migration and homing of AML cells. Meanwhile, MALAT1 activates the Wnt/β-catenin pathway by promoting translocation of β catenin to the nucleus and further facilitates the proliferation of LSCs. Similarly, overexpression of HOTAIR activates PI3K/AKT signaling to promote growth and chemoresistance in AML cells and modulates Wnt/β-catenin signaling through epigenetic modifications. Besides that, HOTAIR represses E-cadherin expression, which promotes epithelial-to-mesenchymal transition, increasing the invasion and migration capabilities of AML cells. Collectively, overexpression of MALAT1 and HOTAIR orchestrates an intricate network of interactions with numerous signaling pathways to try and achieve an AML-favorable microenvironment for its promotion and maintenance.

MALAT1 is associated with the regulation of most of the significant pathways in AML. It significantly modulates the PI3K/AKT pathway mainly implicated in cell survival, proliferation, and resistance to apoptosis. MALAT1 causes the activation of the PI3K/AKT pathway; thus, it results in increased proliferation and survival of AML cells [142]. Therefore, this activation supports persistence in the bone marrow niche of LSCs by inhibiting apoptosis and facilitating cell growth. The upregulation of this pathway also impinges on CXCR4 signaling in that MALAT1 can promote CXCR4 and its ligand CXCL12 expression to facilitate AML cell migration and homing into the bone marrow niche [143]. Meanwhile, MALAT1 regulates the Wnt/β-catenin pathway by interacting with β-catenin, thus facilitating its nuclear translocation that further activates the Wnt target genes. Consequently, this facilitates proliferation of the AML stem cells and promotes disease processes [144]. MALAT1 targeted the MEK/ERK pathway to increase the survival and proliferation of AML cells by modulating differentiation and proliferation (Figure 4). In the context of AML, this pathway is frequently activated by MALAT1 and it drives the expression of genes responsible for cell growth and resistance to therapy [100].

Additionally, single-cell RNA-sequencing (scRNA-seq) studies have revealed that MALAT1 is selectively enriched in AML stem-like cells and niche-interacting subpopulations, further supporting its role in maintaining leukemic cell–stromal interactions [145]. MALAT1 localizes to nuclear speckles and interacts with splicing regulators (e.g., SRSF1), influencing transcriptome remodeling in response to bone marrow niche signals. Furthermore, scRNA-seq-based ligand–receptor interaction analysis highlights MALAT1 as a potential modulator of CXCR4/CXCL12 signaling through indirect upregulation of CXCR4 mRNA stability and suppression of miR-101, a CXCR4 inhibitor. These findings place MALAT1 at the center of leukemia–stromal adhesion, immune evasion, and chemoresistance within the bone marrow microenvironment [146].

HOTAIR also modulates major signaling pathways involved in AML. Like MALAT1, HOTAIR influences the PI3K/AKT pathway, which plays a significant role in the regulation mechanism of cell survival and proliferation [75]. In light of this fact, HOTAIR modulates this pathway through interacting with various signaling components and thus promotes AML cell growth and chemoresistance. Another important pathway that HOTAIR modulates is the Wnt/β-catenin pathway. In this context, HOTAIR collaborates with polycomb repressive complexes in carrying out epigenetic regulation through histone modification or changes in chromatin that, in turn, modulate β-catenin signaling and the expression of Wnt target genes that support the self-renewal and proliferation of AML stem cells [147]. This has been discussed in great detail by Zarkou et al. HOTAIR suppresses the expression of E-cadherin in E-cadherin signaling, thus promoting epithelial-to-mesenchymal transition and facilitating the invasion and migration of AML cells. Downregulation of E-cadherin expression influences further the MEK/ERK pathway, modulating cell adhesion and motility in AML, affecting cell proliferation and survival [148].

HOTAIR, on the other hand, exerts its effects through epigenetic silencing mechanisms involving PRC2 and LSD1 recruitment, which not only repress tumor suppressor genes but also modulate chromatin structure to enable AML clone persistence [149]. Beyond pathway modulation, HOTAIR contributes to clonal fitness and subtype-specific survival by promoting HOXA cluster gene expression in FLT3-ITD and NPM1-mutant AML. These functions highlight its role in sustaining dominant leukemic clones, particularly within therapy-resistant bone marrow niches [150].

MALAT1 and HOTAIR participate in the regulation of interaction between CXCR4 and E-cadherin signaling. The PI3K/AKT and MEK/ERK pathways of these lncRNAs modulate CXCR4 signaling in AML cell migration and homing. MALAT1 regulates CXCR4, which entails retention and migration in the niche, while HOTAIR acts by E-cadherin, promoting disruption of cell–cell adhesion and invasiveness. All these interactions illustrate the complexity of the role that lncRNAs play in shaping the AML microenvironment and sustaining the disease [151, 152].

## 5. Conclusion

In conclusion, the function of long noncoding RNAs, especially MALAT1 and HOTAIR, in AML points out their role as a very important regulator in the molecular landscape of tumors. Their influence runs from several aspects of tumor hallmarks such as cell proliferation, survival, and migration, with an interaction in the bone marrow microenvironment, further promoting AML. These lncRNAs are thus implicated in epigenetic modulation, gene silencing, and critical signaling pathways underlying their use both as biomarkers and therapeutic targets. MALAT1 and HOTAIR further offer complementary insights into AML subtypes with key implications for early diagnosis, prognosis, and treatment response. While this represents a promising avenue, further exploration of the functional roles played by lncRNAs and their multifaceted interactions with AML cells, especially with regard to a more detailed explanation of their role within the bone marrow microenvironment, is required. These will be imperative in future studies that hopefully will translate these findings into clinical applications, which will then contribute to precision medicine and open new avenues of management of AML (Table 1).

## Figures and Tables

**Table 1:** List of previous studies on the roles of HOTAIR and MALAT-1 in AML.

**Previous studies**				
	**Objective**	**Outcome**	**Conclusion**	**Reference**
1	This study examined the association between recurrent mutations and clinical features in elderly patients with cytogenetically normal acute myeloid leukemia (CN-AML) and long noncoding RNAs (lncRNAs). A specialized microarray technique was utilized to evaluate the lncRNA expression profiles in 148 newly diagnosed cases of CN-AML.	The findings revealed a set of 48 lncRNAs that were significantly linked to event-free survival in this older patient group. Comparing those with a lower lncRNA score to those with a higher one, the former showed lower rates of full response, shorter periods of disease-free survival, and poorer overall survival. Subsequent validation analyses supported these results and established the lncRNA score as an independent prognostic marker in multivariable analysis.	In conclusion, while lncRNA expression profiles in AML were found to be associated with frequently occurring mutations, only a limited number of lncRNAs showed a strong correlation with treatment outcomes and patient survival.	[153]
2	This study set out to evaluate the expression levels of HOTAIR in bone marrow samples from individuals who had been diagnosed with acute myeloid leukemia (AML) from scratch.	The results showed that HOTAIR was overexpressed in AML patients in comparison with those with complete remission, and healthy individuals.	In conclusion, our investigation demonstrated the significant overexpression of HOTAIR in AML patients and its correlation with a number of clinicopathological variables that could affect this population's prognosis.	[116]
3	The purpose of this study was to assess the clinical significance of HOTAIR expression in the advancement of tumors and ascertain the expression levels of HOTAIR in patients with acute myeloid leukemia (AML).	Results indicated that the AML patients had a significantly higher overexpression of HOTAIR compared to healthy controls. In addition, higher expression of HOTAIR significantly correlated with higher WBC count and a higher percentage of bone marrow blasts. Moreover, patients with high HOTAIR expression significantly worse survival and relapse-free survival than HOTAIR low expressed patients.	These findings indicate overexpression of HOTAIR in newly diagnosed AML patients, its correlation with leukemic burden, and reduced overall and relapse-free survival. Hence, it may act as a potential prognostic biomarker, and this lncRNA may be targeted in the therapeutic strategies in AML.	[154]
4	In order to delve into HOTAIR's potential molecular marker for leukemia progression and prognosis, the expression levels of HOTAIR and its downstream target genes were assessed in AL patients.	The significant upregulation of HOTAIR was correlated to the poor clinical features in the M5 subtype AML patients. The overall survival and event-free survival rates were significantly lower as compared with those whose levels of HOTAIR expression were low.	These results suggest that HOTAIR expression may further contribute to leukemogenesis through the regulation of DNA and histone methylation processes, and that it is strongly correlated with a poor prognosis in AL patients.	[155]
5	In patients with acute myeloid leukemia, it aimed to look at HOX transcript antisense RNA expression.	Between AML patients and healthy controls, there was no discernible difference in the HOTAIR lncRNA expression levels.	These findings tend to suggest that HOTAIR is not a valid biomarker for AML diagnosis; nevertheless, further research is required to confirm these conclusions.	[156]
6	The expression levels of MALAT1 and its functional role in AML patients were shown, for the first time, in this current study.	The results revealed that patients with higher levels of MALAT1 expression showed significantly higher incidences of M5 compared with healthy controls. M5 patients with overexpressed MALAT1 had poorer OS compared to patients with low levels of this lncRNA. MALAT1 knockdown was associated with attenuated proliferation, cell cycle disturbance in the M5 cell lines represented by U-937 and THP-1, but increased apoptosis.	These findings showed that elevated MALAT1 expression was linked to a poor prognosis in M5 patients and may have an effect on leukemia cells' ability to proliferate and undergo apoptosis, therefore serving as a proof for its potential to become a theranostic marker.	[106]
7	The purpose of its design was to investigate how HOTAIR controls LSC self-renewal.	The results revealed that compared to normal HSPCs, LSCs exhibited much higher levels of HOTAIR expression.	These findings underline the concept that HOTAIR might promote leukemogenesis by enhancing self-renewal of LSCs and hence define it as a potential pharmacologic target in strategies that have been developed for the eradication of LSCs in the treatment of leukemia.	[157]
8	The aim of this study was to investigate the expression of HOX transcript antisense RNA in AML patients with chromosome 12p abnormalities and its association with various clinicopathological features and clinical outcomes. This investigation also delineated the impact of chromosome 12p aberrations on expression of HOTAIR, miRNA-193a, and the c-kit gene considered as targets of HOTAIR in AML.	Where low HOTAIR and c-kit expressions significantly correlated with better overall survival and leukemia-free survival, while for miR-193a high expression was associated with better overall survival; it failed to attain statistical significance in leukemia-free survival.	The observation of this study was that the 12p chromosomal abnormalities in AML patients generally have a poor prognosis. Moreover, HOTAIR, miR-193a, and c-kit were identified through the findings as independent prognostic markers in AML with associated 12p abnormalities, suggesting that these genes may become potential therapeutic targets for new future AML therapy strategies.	[158]
9	The purpose of this study was to investigate at how long noncoding RNA HOTAIR contributes to the development of acute myeloid leukemia, especially focused on the HOXA5 gene methylation regulation.	It has been shown that in AML, HOTAIR is significantly overexpressed and it promotes the methylation of the HOXA5 gene through recruitment of Dnmt3b. Downregulation of HOTAIR expression or reactivation of HOXA5 led to significantly reduced proliferation of AML cells along with induction of apoptosis both in vitro and in vivo experiments.	These findings have accentuated the antitumor role of HOTAIR silencing in AML by revealing that leukemia progression is inhibited through its role in the promotion of HOXA5 promoter demethylation associated with the reduction of Dnmt3b levels.	[159]
10	The present investigation examined HOTAIR's function in the emergence of leukemia multidrug resistance.	Results demonstrated that the expression levels of HOTAIR were significantly higher in drug-resistant leukemia cells and patient samples. Silencing of HOTAIR repressed cell growth, increased apoptosis, and increased the sensitivity of K562/A02 cells to doxorubicin treatment. Expression of P21 and Notch1 was inhibited, phosphorylation of AKT was diminished in drug-resistant cells after silencing of HOTAIR.	These findings show that the lncRNA HOTAIR regulates P21 expression and further impacts the AKT/Notch1 signaling pathway, which both contribute to the formation of MDR in leukemia cells.	[160]
11	This study was performed with the aim of investigating the effects of HOTAIRM1 on HL60 and THP-1 leukemia cell lines' Ara-C resistance, and determining the mechanisms underlying these processes.	The results demonstrated that HOTAIRM1 knockdown inhibited Ara-C-mediated AML cell death by lowering viability, glycolysis, and Wnt/β-catenin pathway activity. Glycolysis and Ara-C sensitivity were recovered upon reactivation of the Wnt/β-catenin axis.	These findings suggested that knockdown of HOTAIRM1 increased the sensitivity of AML cells to Ara-C through modulation of the Wnt/β-catenin/PFKP signaling pathway and may indicate that HOTAIRM1 can be a promising target in overcoming the resistance to Ara-C in AML.	[161]
12	According to this study, patients with acute myeloid leukemia may develop ADM resistance due in part to HOTAIR.	The findings indicated that whilst PTEN displayed a relatively low expression, HOTAIR was significantly overexpressed in the relapsed or refractory AML patients. Comparing HL60/ADM cells to normal HL60 cells, comparable patterns were found. Downregulation of HOTAIR or overexpression of PTEN increased sensitivity to ADM and enhanced apoptosis, while cells co-transfected with both did not show such phenomena. Besides, the study also revealed that the DNMT3b was also upregulated by HOTAIR, which thus induced the methylation and downregulated PTEN expression	Based on the available data, it appears that HOTAIR increases DNMT3b, which methylation-represses PTEN expression	[162]
13	The current study examined the expression levels of long noncoding RNA HOTAIR in AML patients and linked the latter to a number of clinical characteristics, laboratory parameters, mutations of the FLT3-ITD and NPM1 genes, and treatment outcomes.	The findings showed that AML patients had much higher levels of HOTAIR expression than the control group, and that these levels varied with chemotherapy. HOTAIR expression has been related to factors like hemoglobin concentration, age, total white blood cell count, and presence of NPM1 mutation. What is more, it is related to relapse-free survival among studied patients.	Taken together, these findings suggest that HOTAIR was significantly overexpressed in AML patients and its expression before and after chemotherapy may act as a useful marker for chemosensitivity and recurrence.	[163]
14	Currently, this work examined HOTAIR's role in AML cell differentiation and its potential as a therapeutic target, particularly in individuals with AML who are not APL.	For the first time, this study identified that C/EBPβ-driven upregulation of HOTAIR occurred during ATRA-induced differentiation of AML cells. It was observed that high expression of HOTAIR was related to facilitated differentiation, which induced G1 phase arrest by affecting the p21 through miR-17-5p. Surprisingly, AML patients had significantly lower expression of HOTAIR; in addition, their levels were inversely related to platelet counts in the M2 subtype, thus pointing toward a specific role in this particular subtype.	These findings underlined the possibility of targeting HOTAIR for therapeutic intervention against AML, especially the M2 subtype of AML.	[164]
15	The purpose of this study was to investigate the expression of lncRNA HOTAIR in newly diagnosed AML patients' bone marrow mononuclear cells prior to their initial chemotherapy treatment. The aim is to find a diagnostic marker that could be used for its correlation with certain classic prognostic factors.	A *p* value of 0.000 statistically established that the expression of HOTAIR was substantially higher in AML patients based on these data. ROC analysis also indicated that AML patients were clearly distinguishable from healthy controls, hence reinforcing HOTAIR as a diagnostic biomarker. The expression levels of HOTAIR did not significantly correlate with event-free survival or any other prognostic indicator.	The study concluded that although highly overexpressed in de novo AML cases, HOTAIR is a promising diagnostic marker; however, its overexpression does not relate to an unfavorable prognosis.	[117]
16	The purpose of this study was to evaluate the expression of miR-143 in AML patients and its impact on the growth and apoptosis of AML cells. This study also intended to explore the interaction between miR-143 and long noncoding RNA MALAT1 in order to find the possible regulating mechanism of miR-143 in the progression of AML.	In the present study, the expression levels of miR-143 in AML patients were significantly lower than those in healthy controls. The induced overexpression of miR-143 triggered prominent cell growth inhibition and induced apoptosis in U-937 cells. Additionally, the authors identified that miR-143 could directly bind with the MALAT1 RNA sequence, which repressed MALAT1 expression in U-937 cells, whereas silence of MALAT1 had no obvious effect on miR-143 expression.	These findings of downregulation of miR-143 in AML patients suggest that overexpression contributes to the suppression of cell growth and induction of apoptosis at least partially through interaction with MALAT1.	[165]
17	This work was designed to investigate the role of MALAT1 in AML patients and further explore the regulatory network formed by MALAT1, miR-146a, and CXCR4.	The expression of MALAT1 and CXCR4 were obviously higher in AML. The knockdown of MALAT1 decreases the migratory and proliferatory capabilities of cells, inducing apoptosis. The present study suggested that MALAT1 acts as a ceRNA and downregulated miR-146a expression, which in turn controls the behavior of the cells by directly targeting CXCR4. MALAT1 silencing decreased CXCR4 levels, which were partially reverted upon miR-146a inhibition.	The findings of this study suggest that MALAT1 modulates AML cell migration, proliferation, and apoptosis by directly interacting with miR-146a targeting CXCR4 expression. Thus, MALAT1 is a promising candidate for the development of therapeutic options against AML.	[146]
18	The objective of this research was to examine the epigenetic processes by which the long noncoding RNA MALAT1 represses the E-cadherin gene in individuals with acute myeloid leukemia.	Results demonstrated that MALAT1, EZH2, and EED were highly expressed in AML patients with low expression levels of E-cadherin. Besides, it was indicated that there was a positive correlation between the expression levels of MALAT1 and the expression of EZH2 and SUZ12. On the contrary, MALAT1 expression knockdown increased the levels of E-cadherin and reduced the expression levels of some epigenetic factors.	This infers that MALAT1 might take part in the E-cadherin gene silencing through recruiting EZH2 and SUZ12, which in turn mediates H3K27 trimethylation—an epigenetic mark for transcriptional repression.	[166]
19	The expression of lysine methyltransferase 2A in acute myeloid leukemia cells was the main focus of this investigation, which also looked at the underlying molecular mechanisms that may affect the proliferation of AML cells.	The results indicated that the PCR signals in the KMT2A-expressed group were far stronger than that of the control group. Additionally, the number of EdU + cells was obviously higher in both KMT2A-OE and HOTAIR-OE groups and showed a declining trend in KMT2A-KD and HOTAIR-KD groups. Besides, p-Akt and p-mTOR levels in KMT2A-OE and HOTAIR-OE groups are higher as compared to the other three groups (*p* < 0.01).	These results suggest that in order to maintain AML cells, KMT2A interacts with the long noncoding RNA HOTAIR to activate the Akt/mTOR signaling axis.	[167]
20	The purpose of this work was to evaluate the relationship between the differential expression of FLT-3, c-Myc, STAT3, STAT5, and p27 in AML patients and the differential expression of specific lncRNAs, such as HOTAIR, PVT-1, and CRNDE.	The results showed that among AML patients, the expressions of FLT3, c-Myc, STAT3, and HOTAIR were upregulated, but the expression of p27 was noticeably downregulated. It was shown that FLT3, through inhibition of p27 via a c-Myc-dependent pathway, may enhance the proliferation and survival of AML cells. Furthermore, overexpression of HOTAIR was indicated to upregulate STAT3 and promote proliferation in leukemia cells.	The genes HOTAIR, FLT-3, c-Myc, STAT3, and p27 stand out as possible biomarkers for AML patients and as novel targets for therapeutic intervention based on the results of the gene expression study.	[168]
21	Investigating MALAT1's expression level and functional relevance in patients with acute myeloid leukemia was the goal of this investigation.	The study found that the expression of MALAT1 was notably higher in AML patients. MALAT1 suppression resulted in lower levels of proliferation, migration, and invasion with higher apoptosis rates in AML cells. Further investigation revealed that MALAT1 interacts with METTL14 to increase the m6A modification of ZEB1 and that overexpression of ZEB1 can partly rescue the effect caused by MALAT1 inhibition.	These results therefore imply that MALAT1 is essential for controlling the m6A alteration of ZEB1, which in turn promotes the aggressive features of AML.	[108]
22	This research examined the impact of exosomes derived from BM-MSCs on cellular and molecular properties in HL-60 cells, an in vitro model cell line of AML. It researched the ability of these exosomes to regulate the expression of lncRNAs associated with the progression, proliferation, and drug resistance of AML, and of those with poor prognosis.	The findings indicated that BM-MSC exosomes inhibited the HL-60 cells' ability to proliferate, undergo metabolic processes, and go through the cell cycle, which was accompanied by the induction of ROS levels leading to increased apoptosis. This was linked to decreased levels of BCL2, c-Myc, MALAT1, HOTAIR, and H19 and increased expression of p53, p21, BAX, and FOXO4.	Overall, BM-MSC-derived exosomes may offer a new adjuvant in the therapy of leukemia.	[169]

## Data Availability

Data sharing is not applicable to this article as no new data were created or analyzed in this study.

## Nomenclature

CN-AML: Cytogenetically normal acute myeloid leukemia
LncRNAs: Long noncoding RNAs
HOTAIR: HOX transcript antisense intergenic RNA
AML: Acute myeloid leukemia
WBC: White blood cells
DNA: Deoxyribonucleic acid
RNA: Ribonucleic acid
AL: Acute leukemia patients
MALAT1: Metastasis-associated lung adenocarcinoma transcript 1
U-937: Uppsala 937 human histiocytic lymphoma cell line
THP-1: Tsuchiya human monocytic leukemia cell line 1
LSC: Leukemia stem cell
miRNA-193a: MicroRNA-193a
c-kit gene: KIT proto-oncogene, receptor tyrosine kinase
AKT: v-Akt murine thymoma viral oncogene homolog
Notch1: Neurogenic locus notch homolog protein 1
ADM resistance: Adriamycin resistance
MDR: Multidrug resistance
HOTAIRM1: HOXA transcript antisense RNA myeloid-specific 1
HL60: Human acute promyelocytic leukemia cell line
Ara-C: Cytarabine
Wnt/β-catenin pathway: Wingless/integrated (Wnt) signaling pathway and β-catenin transcriptional co-activator
PFKP: Phosphofructokinase, platelet type
PTEN: Phosphatase and Tensin Homolog deleted on chromosome Ten
DNMT3b: Methyltransferase 3 beta
FLT3-ITD: FMS-like tyrosine Kinase 3–internal tandem duplication
NPM1: Nucleophosmin 1
APL: Acute promyelocytic leukemia
ROC: Receiver operating characteristic
CXCR4: C-X-C chemokine receptor Type 4
ceRNA: Competing endogenous RNA
EZH2: Enhancer of zeste Homolog 2
EED: Embryonic ectoderm development
SUZ12: Suppressor of Zeste 12
H3K27: Histone H3 Lysine 27
EdU + cells: 5-Ethynyl-2′-deoxyuridine positive cells
KMT2A-OE: Lysine methyltransferase 2A overexpression
Akt: Protein kinase B
mTOR: Mechanistic target of rapamycin
c-Myc: Cellular myelocytomatosis oncogene
STAT3: Signal transducer and activator of Transcription 3
STAT5: Signal transducer and activator of Transcription 5
PVT-1: Plasmacytoma variant Translocation 1
CRNDE: Colorectal neoplasia differentially expressed
ZEB1: Zinc finger E-box binding Homeobox 1
METTL14: Methyltransferase-like 14
BM-MSCs: Bone marrow–derived mesenchymal stem cells
BCL2: B-cell Lymphoma 2
BAX: BCL2-associated X, apoptosis regulator
FOXO4: Forkhead box O4
